# Comparative evaluation of *Saccharomyces*, *Bacillus*, and *Lactobacillus* as antibiotic alternatives on growth performance, redox balance, and gut microbiota in broilers

**DOI:** 10.1038/s41598-026-65377-9

**Published:** 2026-08-13

**Authors:** Ahmed Samy, Hoda Kabary, Hany M. R. Elsherif

**Affiliations:** 1 Department of Animal Production, National Research Centre, Cairo, 12622 Egypt; 2 Agricultural Microbiology Department, National Research Centre, Cairo, 12622 Egypt; 3 Animal Production Department, Faculty of Agriculture, Cairo University, Giza, Egypt

**Keywords:** Antioxidant status, Broiler performance, Gut microbiota, Probiotics, Biotechnology, Microbiology

## Abstract

This study evaluated the efficacy of *Saccharomyces cerevisiae* (SC), *Bacillus subtilis* (B), *Lactobacillus casei* (LB), and their combination (Mix) as natural alternatives to antibiotics in broiler nutrition. Liquid cultures were prepared to a density of 10⁸ CFU/mL and examined morphologically by light microscope and scanning electron microscopy. Three hundred one-day-old Arbor Acres broiler chicks were randomly allocated to five groups: a control (basal diet) and four groups receiving 900 ppm of B, LB, SC, or Mix from day 11 to day 33, using a completely randomized design with six replicates per treatment. Growth performance, antioxidant indices, thyroid hormones, and intestinal microbiota were assessed. Dietary supplementation with B, LB, SC, or Mix significantly improved body weight gain and feed conversion ratio compared with the control. In addition, enhanced total antioxidant capacity and catalase activity, along with reduced MDA levels, indicated improved oxidative status. Probiotic supplementation increased intestinal *Lactobacillus* counts, suppressed Salmonella spp., and reduced total bacterial count. Moreover, no adverse effects were observed on thyroid hormones or liver and kidney function indicators. These findings suggest that the tested probiotics are effective antibiotic alternatives for improving broiler health, productivity, and gut microbial balance. The combined probiotic treatment improved several evaluated parameters, supporting sustainable poultry production practices.

## Introduction

Antibiotics have been prohibited from being utilized as growth stimulants in broilers; instead, probiotics, plant extracts, and organic acids have been used^1–6^.

Probiotics are live microorganisms which, when administered in adequate amounts, confer a health benefit on the host and are widely used as feed additives in poultry production^7^. They have emerged as promising feed additives for broiler production, particularly in response to restrictions on the use of antibiotic growth promoters^7^.

There are numerous ways in which probiotics might benefit the digestive system, including enhancing nutrient digestibility, regulating the microbiota, enhancing the barrier function of the digestive tract, minimizing gastrointestinal disorders, and improving the immune system^7,8^. Also, probiotics enhance gastrointestinal function by lowering pH, and improving the activity of enzymes in the gastrointestinal tract and intestinal microbiota^9^.

Probiotics have been proven to promote broiler growth and feed efficiency, as well as enhance the immune response and protect chicks from diseases, in addition to improving intestinal villi histomorphology and intestinal microbiota^10–13^. Soomro et al.^14^ concluded that broiler diets supplementing with probiotics improves carcass yield, weight gain, and immunological state^15^. It could assist broilers fed antibiotic-free diets improve antibody titers against infectious bursal disease^13^.

*Bacillus subtilis* is recognized for its spore-forming ability, enzyme production, and stability during feed processing^16^. *Lactobacillus casei* contributes to maintaining intestinal microbial balance and inhibiting pathogenic bacteria through organic acid production^17^. *Saccharomyces cerevisiae* has been reported to improve gut health, nutrient utilization, and antioxidant status through its yeast-derived bioactive components^18^.

Despite the growing body of evidence, direct comparisons among *Bacillus subtilis*, *Lactobacillus casei*, *Saccharomyces cerevisiae*, and their combination under identical experimental conditions remain limited. It was hypothesized that dietary supplementation with these probiotics, either individually or in combination, would improve growth performance, redox balance, and gut microbiota in broiler chickens. Therefore, this study aimed to compare their effects on these parameters.

## Materials and methods

### Ethical approval

All animal handling and experimental procedures were approved by the Medical Research Ethics Committee of the National Research Centre (Approval No. 13050405). The study was conducted in accordance with the journal’s ethical policies, the European Union regulations for the protection of animals used for scientific purposes, and the ARRIVE guidelines (https://arriveguidelines.org/). At the end of the experimental period, birds selected for sampling were humanely slaughtered by trained personnel using standard poultry processing procedures designed to minimize stress and suffering prior to tissue and blood collection.

### Birds, housing and management

This experiment was conducted at the Poultry Nutrition Research Unit (PNRU), Faculty of Agriculture at Cairo University, Giza, Egypt. Three hundred Arbor Acres broiler chicks were grown in a warmed brooder house for 10 days before being randomly housed at semi-close system in three-deck batteries and divided into 5 groups, each with 60 chicks separated in 6 replicates from 11 to 33 days of age.

Throughout the experimental trial, light was provided for 16 h /day. Water and food were available at all times. Birds received a program against the avian flu, Infectious bronchitis, and Infectious bursal disease, the New Castle virus.

### Experimental materials

#### Liquid cultures preparations and stock feeds mixture addition

Fresh cultures of *Lactobacillus casei* (LB), *Bacillus subtilis* (B) and *Saccharomyces cerevisiae* (SC), were prepared in De Man–Rogosa–Sharpe (MRS), nutrient broth (NB) and Potato dextrose broth (PDB) liquid medium respectively. The strains were purified and identified at Agricultural, Microbiology Department, National Research Centre, Cairo, Egypt. The incubation condition for *L. casei* were 3 days at 37 °C in tightly closed 250 ml glass bottles under static, microaerophilic conditions, while *B. subtilis* and *S. cerevisiae* were kept in shaker incubator at 30 °C for 48 h at 180 rpm. Reaching O.D 600: 0.5–1 nm (10^8^ CFU), an equal volume of calcium carbonate was added to each culture (1:1w/v) and left for complete drying in oven at 40 °C. calcium carbonated mixed cultures were then ground aseptically and 1 g of each mixed culture were evaluated on MRS, NA and Potato dextrose agar (PDA) agar plates to ensure desired count before adding to the basal diet using serial dilution plate count method. Light microscope and Field emission scanning electron microscope were used to morphological identification of each sample. The viable count of each dried probiotic preparation was approximately 1 × 10⁸ CFU/g, as determined by the serial dilution plate count method. The probiotic preparations were incorporated into the basal diet at 0.9 g/kg feed (900 ppm), corresponding to a final dose of approximately 9 × 10⁷ CFU/kg feed. For the mixed-probiotic treatment, each strain was added at 0.3 g/kg feed, providing approximately 3 × 10⁷ CFU/kg feed per strain (total 9 × 10⁷ CFU/kg feed).

#### Experimental design and diets

The chicks were randomly classified into 5 groups, 6 replicates of 10 chicks each. The group one (control) was received the basal grower and finisher diets according to Arbor Acres nutrient specifications (Table 1). Diets were supplemented with probiotic preparations at 0.9 g/kg feed (900 ppm). Groups 2, 3, and 4 received B. subtilis, L. casei, or S. cerevisiae, respectively, whereas Group 5 received a mixture containing 0.3 g/kg feed of each strain (300 ppm each).

**Table 1:** Formulation and nutrients composition of the starter, grower and finisher diets.

Ingredients %	Period	
	Grower (11–25 days)	Finisher (26–33 days)
Yellow maize	56.03	63.00
46% Soybean meal	31.20	25.13
Gluten meal (60%)	5.30	5.00
Soybean oil	3.50	3.10
Limestone	1.04	0.93
Mono calcium phosphate	1.60	1.58
Common salt (NaCl)	0.20	0.20
NaHCO_3_	0.23	0.23
Vitamin & Mineral mix^(1)^	0.30	0.30
DL-methionine	0.14	0.13
L-lysine HCl	0.32	0.28
Threonine	0.09	0.07
Choline chloride	0.05	0.05
Total	**100**	**100**
Calculated composition^(2)^		
Crude protein %	21.50	20.0
ME (Kcal/kg)	3100	3200
Ether extract%	6.01	5.87
Crude fiber%	3.49	2.43
Lysine %	1.29	1.19
Methionine%	0.51	0.51
Methionine + Cystine %	0.88	0.87
Threonine%	0.88	0.81
Calcium %	0.87	0.81
Nonphytate P %	0.435	0.405
Sodium%	0.16	0.16
Chloride%	0.16	0.16

^(1)^Vitamin - mineral mixture supplied per Kg of diet: Vit A, 12,000 IU; Vit E, 10 mg Vit D_3_, 2200 IU;; Vit K_3_, 2 mg; Vit B_1_, 1 mg; Vit B_6_, Vit B_2_, 4 mg; 1.5 mg; Vit B_12,_ 10 µg; Pantothenic acid, 10 mg; Niacin, 20 mg; Folic acid, 1 mg; Biotin, 50 µg; Choline chloride, 500 mg; Iodine, Copper, 10 mg; 1 mg; Iron, 30 mg; Selenium, 0.1 mg; Zinc, 50 mg and Manganese, 55 mg.

^(2)^According to NRC 1994.

### Measured parameters

#### Productive performance parameters

Body weight; all chicks were weighted at 25 (grower stage) and 33 days old (finisher stage) to calculate the average individual live body weight and average weight gain (WG). In addition, the average feed consumption (FC, g/chick) was calculated at 25 and 33 days old. Feed conversion ratios (FCR) were calculated via dividing FC / WG for grower, finisher and overall periods^2^.

#### Intestinal microbiota

At 33 days of age, 5 birds per each treatment were slaughtered and cecal contents were collected. One gram of the broiler cecum was suspended in double distilled water (DDH2O) and serial dilutions were made till dilution factor 10^8^. Probiotic *Lactobacillus* count were detected on MRS agar plates, while total fecal bacteria and Salmonella count were evaluated on MacConkey, Salmonella-Shigella (SS) agar plates respectively for 3days 37 °C.

#### Blood constituents

At 33 days of age, 5 chicks per treatment were fasted for 6 h and samples of blood were collected through the vein. The blood samples were centrifuged for 10 min at 2000 rpm and the serum was stored for later analysis. Blood serum analysis was conducted using commercial kits (Bio Diagnostic, Cairo, Egypt) in spectrophotometer with a high-performance (FlexorEL200 Biochemical Analyzer) for Aspartate aminotransferase (AST), Alanine aminotransferase (ALT), total protein, albumin (Alb), and alkaline phosphatase (ALP) and urea. ELISA kits (My Bio Source Inc, San Diego, CA) were used to test total Triiodothyronine (T3) and Tetraiodothyronine (T4) hormone in blood serum, and the T3/T4 ratio was computed. Total antioxidant capacity (TAC), catalase (CAT), and malondialdehyde (MDA) levels in serum were measured to determine antioxidant status.

#### Statistical analysis

The General Linear Model of SAS^19^ was used to perform statistical analysis (one-way analysis of variance). Duncan’s new multiple range test (*P* < 0.05) was used to distinguish significant differences between treatment averages^20^.

  

$${{\mathrm{Y}}_{{\mathrm{ij}}}}=\mu +{{\mathrm{T}}_{\mathrm{i}}}+{{\mathrm{E}}_{{\mathrm{ij}}}}$$

where Y_ij_ is the observed value (the j^th^ observation on the i^th^ treatment), µ is the overall mean, T_i_ is the fixed effect of the _i_^th^ treatment, and E_ij_ represents the random error.

## Results

### The morphological structure of the prepared bacteria and fungi

**Fig. 1:**
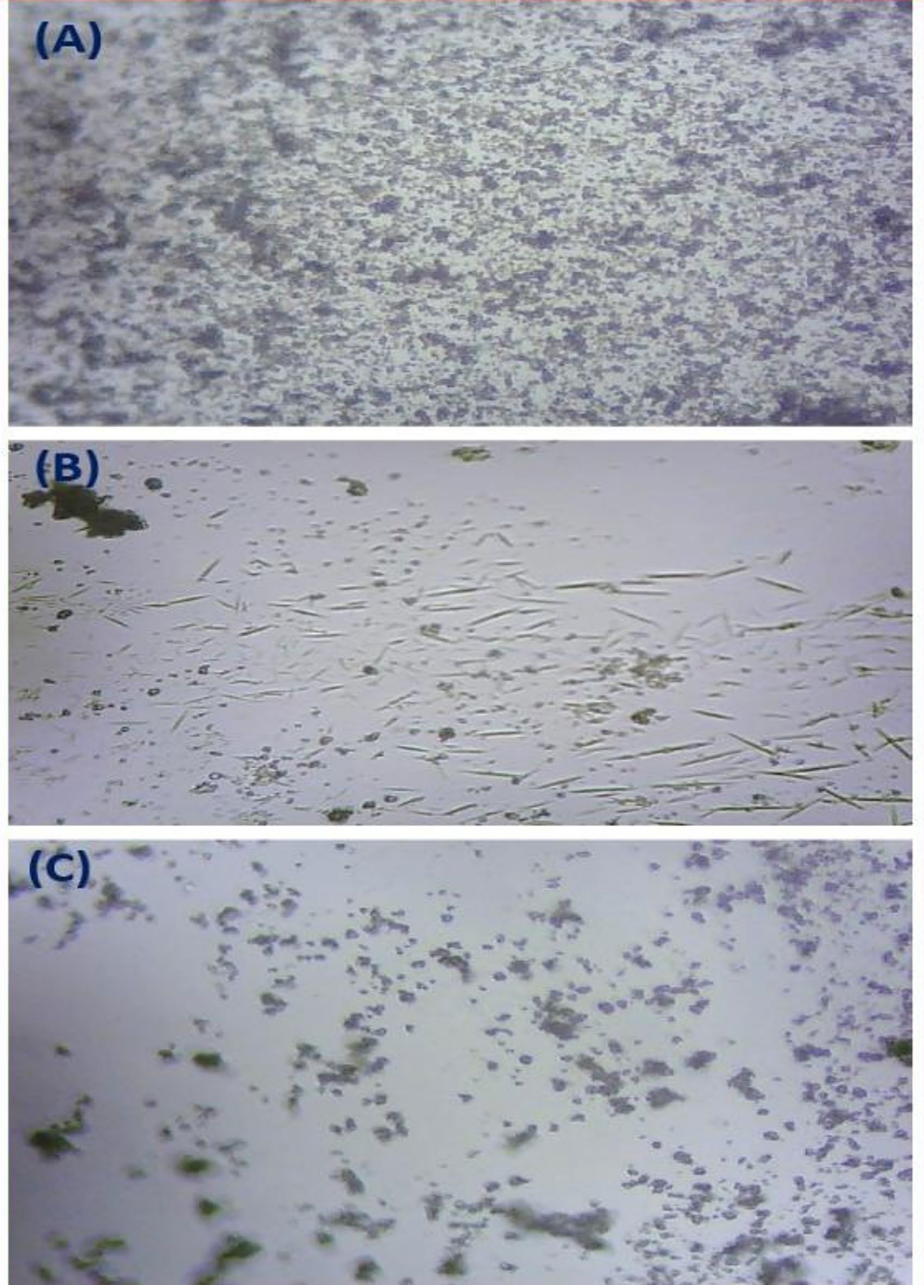
Light Microscope images represents (**A**) *Bacillus subtilis* rod cells impeded with CaCO_3_ particles, (**B**) *Lactobacillus casei* bacterial cells surrounded with CaCO_3_ and (**C**), *Saccharomyces cerevisiae* cells with CaCO_3_.

**Fig. 2:**
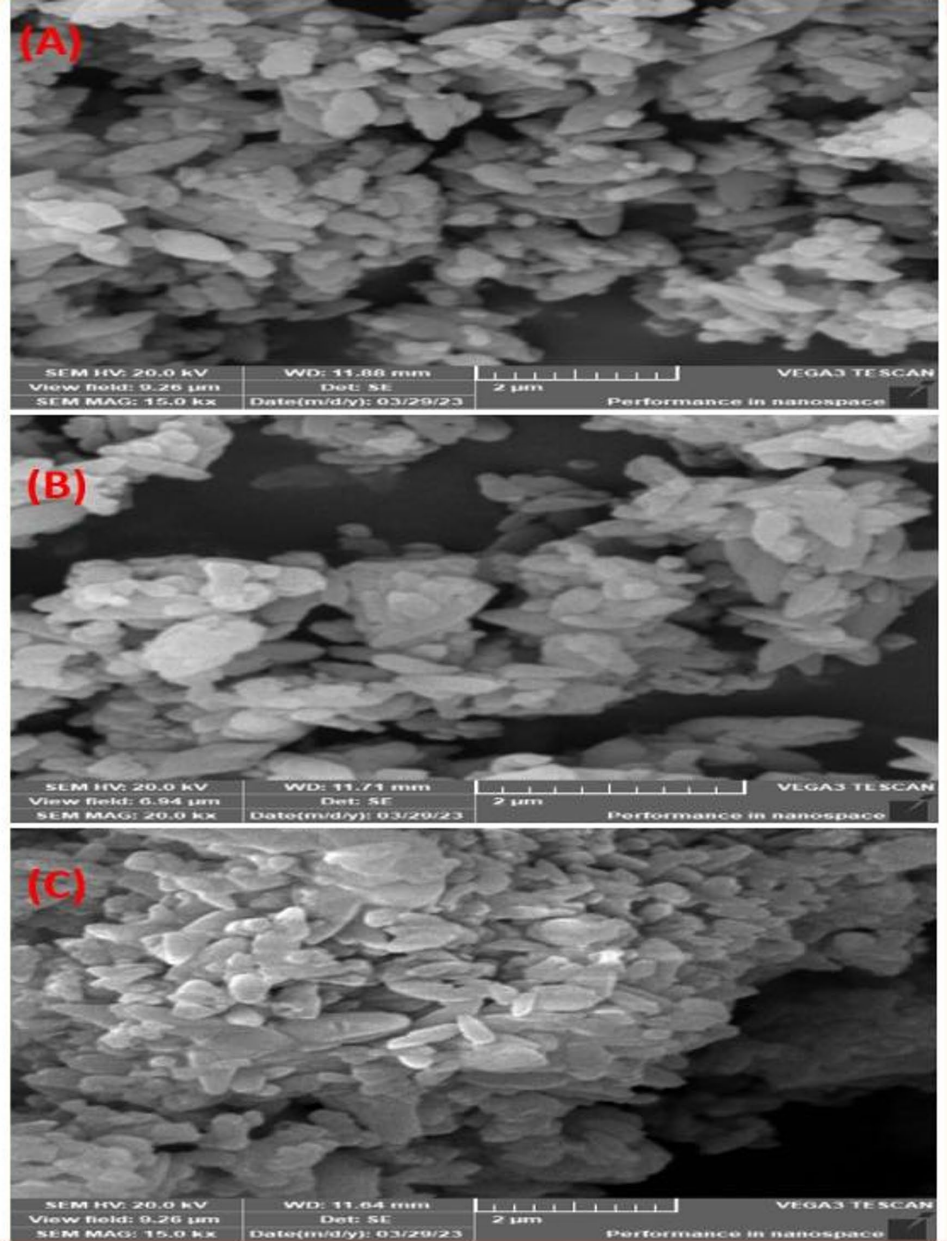
Field emission scanning electron microscope images represents (**A**) *Bacillus subtilis* rod cells impeded with CaCO_3_ particles, (**B**) *Lactobacillus casei* bacterial cells surrounded with CaCO_3_ and (**C**), *Saccharomyces cerevisiae* cells with CaCO_3_.

The morphological structure of the bacteria and fungi that prepared and loaded on the calcium carbonate, *B. subtilis* (Fig. 1A), *Lactobacillus casei* (Fig. 1B), or *S. cerevisiae* (Fig. 1C), is shown in Figs. 1 and 2. Light microscopes and Field emission scanning electron microscope (FESEM) images of the bacteria showed the rod-shaped bacterium in between the particles of CaCO_3_. Similarly, the FESEM image showing sprouting semicircular cells stuck within CaCO_3_ particles was obtained by immersing the *S. cerevisiae* growing culture in the same weight to volume amount of CaCO_3_ powder.

### Productive performance

The influence of *Bacillus Sp.* (B), *Lactobacillus Sp.* (LB), *Saccharomyces cerevisiae* (SC) and their mixture (Mix) on the productive performance of broiler chicks 11–33 days of age are presented in Table 2.

**Table 2:** Effect of dietary treatments on productive performance of broiler chicks from 11 to 33 days old.

Stage	Parameter	Treatments						
		Control	*Bacillus subtilis*	*Lactobacillus casei*	*Saccharomyces cerevisiae*	MIX	SE of means	Significances
Grower (11–25 day)	Beginning weight (g)	306.8	310.8	311.5	312.7	306.3	1.20	NS
	Body weight gain (g)	1172.6^b^	1291.8^a^	1304.4^a^	1241.8^ab^	1260.1^a^	15.41	*
	Feed intake (g/chick)	1871.1	1907.7	1902.7	1883.3	1863.3	8.23	NS
	FCR g feed/ g gain	1.60^a^	1.48^b^	1.46^b^	1.52^ab^	1.48^b^	0.02	*
Finisher (26–33 day)	Body weight gain (g)	397.3^b^	451.9^ab^	488.5^a^	469.6^ab^	490.8^a^	13.42	*
	Feed intake (g/chick)	682.7	737.3	746.7	693.3	697.0	16.23	NS
	FCR g feed/ g gain	1.72^a^	1.64^ab^	1.52^bc^	1.48^c^	1.43^c^	0.03	**
Overall (11–33 day)	Body weight gain (g)	1569.9^b^	1743.7^a^	1792.9^a^	1711.3^a^	1750.9^a^	23.94	**
	Feed intake (g/chick)	2553.8	2645.0	2649.3	2576.7	2560.3	19.60	NS
	FCR g feed/ g gain	1.63^a^	1.52^b^	1.48^b^	1.51^b^	1.46^b^	0.02	**

*p < 0.05, **P < 0.01, and NS: Not significant (*P* > 0.05) do not show a statistically significant difference between means marked with the same letter within the same row.

In grower phase (11-25d), the diets supplementation with B, LB and Mix significantly (*p* < 0.05) enhanced weight gain (WG) and feed conversion ratio (FCR) compared with the control.

In finisher period (26-33d), the birds fed diets inclusion with LB and Mix have the best (*p* < 0.05) WG, whereas FCR was improved for birds fed diets with LB, SC and Mix.

The overall period (11-33d), the addition of B, LB, SC and Mix gave the superior (*p* < 0.05) WG and FCR compared with control group.

While, feed consumption was not significantly (*p* > 0.05) affected through all experimental periods among all treatments.

### Blood constituents

The influence of different treatments on total protein (TP), albumin (Alb), globulin (G), Alb/G ratio, alkaline phosphatase (ALP), Urea, aspartate aminotransferase (AST), alanine aminotransferase (ALT) of broilers at 33 days old are tabulated in Table 3.

**Table 3:** Effect of dietary treatments on blood constituents at 33-day of age.

Item	T. protein (g/dl)	Albumin (g/dl)	Globulin (g/dl)	A/G ratio	ALP (IU/L)	Urea (mg/dl)	ALT (U/L)	AST (U/L)
Ctrl	2.49^b^	1.33	1.17^b^	1.14^a^	146.80	2.65	13.57	202.67
*Bacillus subtilis*	3.15^a^	1.41	1.74^a^	0.81^b^	147.01	2.55	13.07	204.33
*Lactobacillus casei*	3.30^a^	1.41	1.89^a^	0.75^b^	147.09	2.64	13.40	198.00
*Saccharomyces cerevisiae*	3.32^a^	1.27	2.05^a^	0.62^b^	147.04	2.56	13.53	201.67
Mix	3.50^a^	1.39	2.11^a^	0.66^b^	147.94	2.53	13.43	200.67
SE of means	0.11	0.02	0.13	0.05	0.48	0.06	0.15	1.55
Significances	***	NS	**	**	NS	NS	NS	NS

Means designated with the same letter within the same column are not significantly different at 0.05 level of probability, **P < 0.01,***P < 0.001, NS: Not significant (*P* > 0.05).

**Table 4:** Effect of dietary treatments on total main bacteria count at 33-day of age.

Item	Total fecal coliforms	Lactobacilli	Salmonella sp.
Ctrl	5 × 10^8^	3 × 10^6^	7 × 10^2^
*Bacillus subtilis*	3 × 10^6^	3.5 × 10^8^	Not detected
*Lactobacillus casei*	5 × 10^6^	3 × 10^8^	Not detected
*Saccharomyces cerevisiae*	5 × 10^7^	2 × 10^8^	Not detected
Mix	1 × 10^6^	3 × 10^8^	Not detected

The immune response of chicks fed diets supplemented with B, LB, SC and mix were significantly (*p* < 0.05) enhanced via improving the values of TP, G and Alb/G ratio compared with control. Whereas, no negative effects (*p* > 0.05) were showed among all treatments in bone, kidney and liver functions.

### Antioxidants capacity

**Fig. 3:**
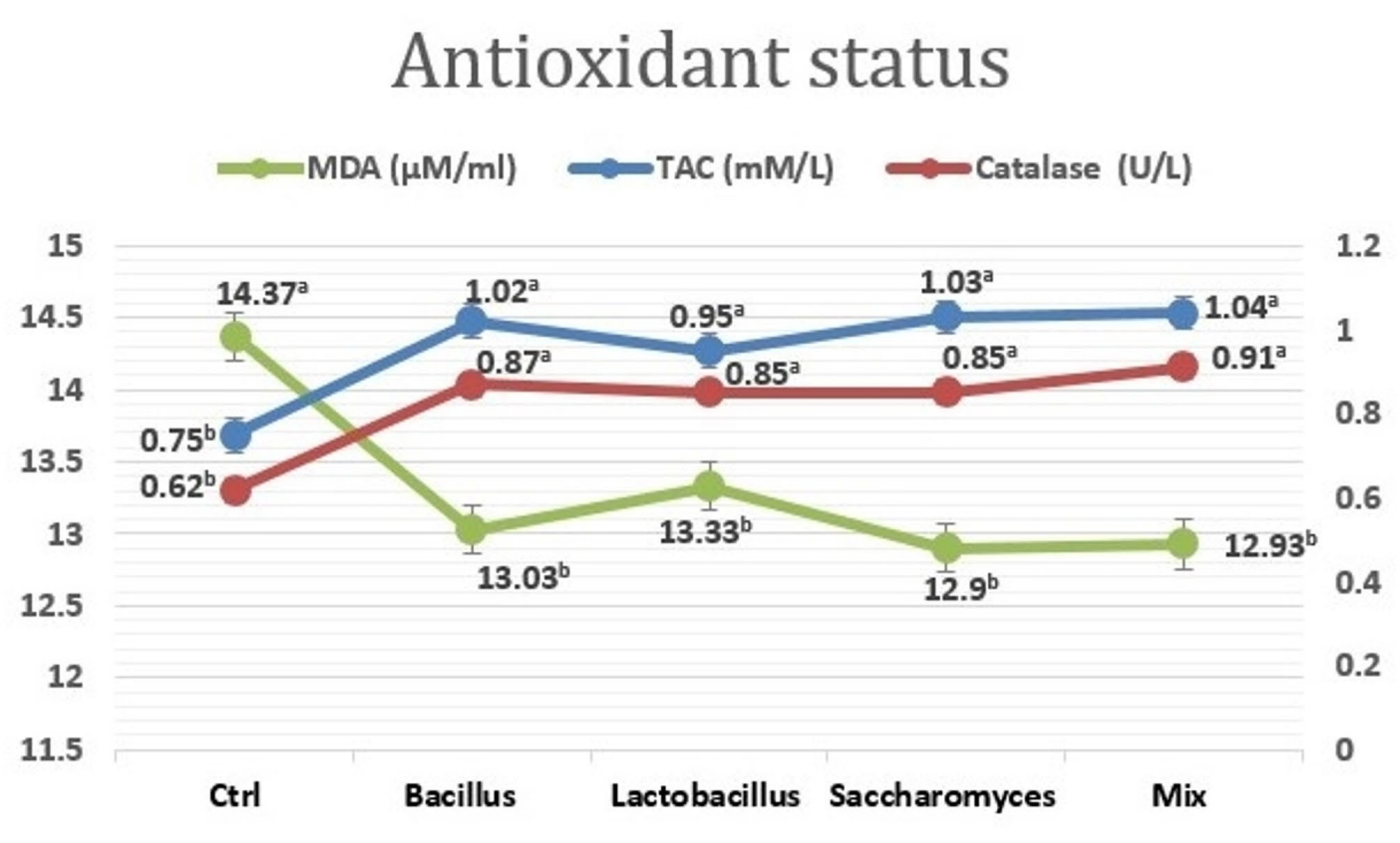
Influence of dietary treatments on antioxidants status at 33-day of age.

Figure 3 shows the influence of different treatments on total antioxidant capacity (TAC), catalase (CAT) and malondialdehyde (MDA) values of broiler chicks at 33 days old.

The values of TAC, CAT and MDA were significantly (*p* < 0.05) improved in B, LB, SC and Mix groups compared with the control, indicating improved antioxidant status.

**Fig. 4:**
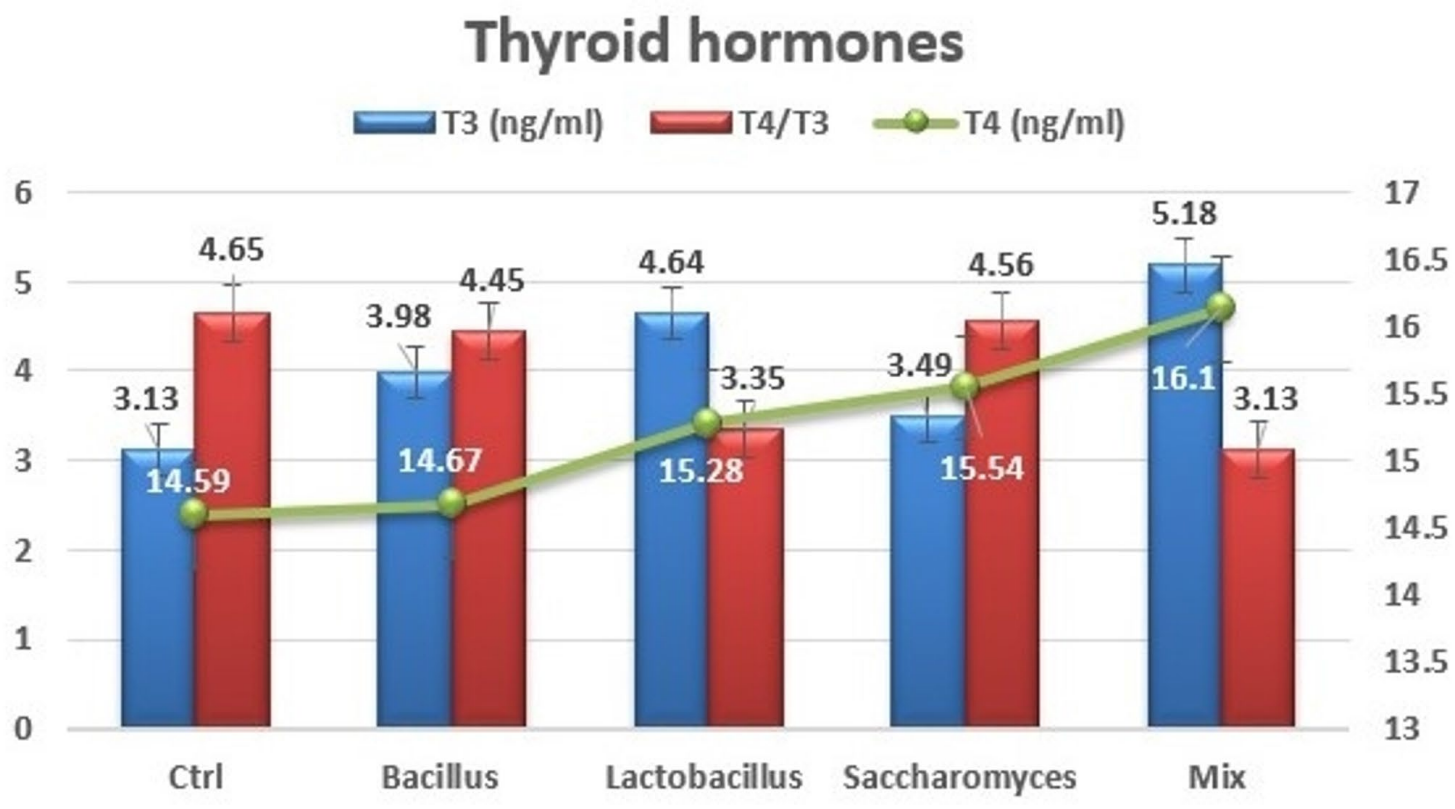
Influence of dietary treatments on thyroid hormones at 33-day of age.

The values of Triiodothyronine (T3), Tetraiodothyronine (T4) and T3/T4 ratio are presented in Fig. 4. No significant differences due to experimental treatments were noticed on either Triiodothyronine (T3) and Tetraiodothyronine (T4) or T3/T4 ratio. No significant differences were observed among treatments in T3, T4, or T3/T4 ratio.

**Fig. 5:**
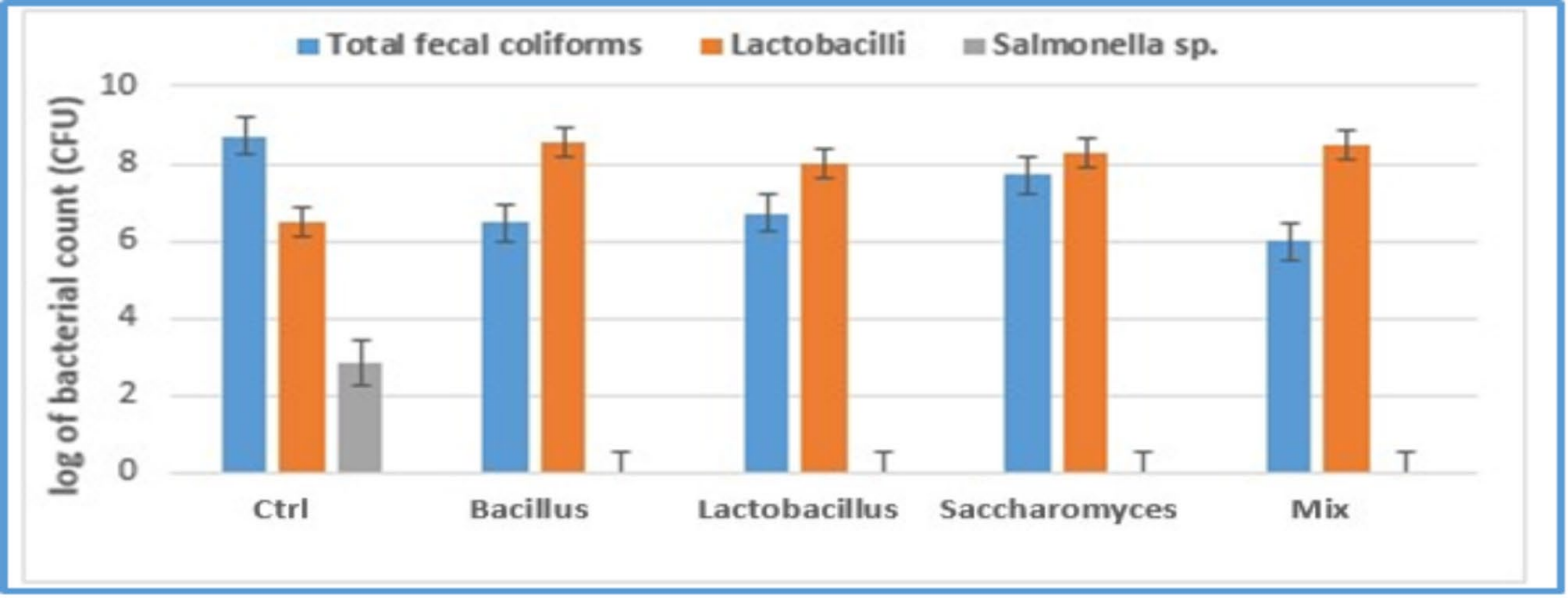
Log of CFU of main bacterial population count on broiler cecum at 33-day of age.

Results in Table 4 and Fig. 5 are representing CFU of the main bacterial genera in 1 g of cecum broilers of after 4weeks feeding on basal diet supplemented with *Lactobacillus casei*, *Bacillus subtilis*, *Saccharomyces cerevisiae* or their mixture. Compared with the control, probiotic treatments increased total *Lactobacillus* counts and decreased total bacterial counts, while *Salmonella* was not detected under the culture conditions used. The most effective feed additive was mixture from the three probiotics added to basal diet that resulted in increase in *Lactobacillus* count (3 × 10^8^) and decrease in total fecal bacteria count (1 × 10^6^).

According to the obtained results, supplementation of *Bacillus Sp.* (B), *Lactobacillus Sp.* (LB), *Saccharomyces cerevisiae* (SC), and their Mixture (Mix) to broiler diets significantly improved (*p* < 0.05) weight gain (WG), feed conversion ratio (FCR), antioxidants state, and intestinal microbiota from 11 to 33 days of age compared to the control, with no adverse effect (*p* > 0.05) on thyroid hormones, bone, liver, and kidney functions.

## Discussion

The increasing restrictions on the use of antibiotic growth promoters in poultry production have encouraged the development of safe and effective alternatives^1–5^, among which probiotics have received considerable attention. In the present study, dietary supplementation with *Bacillus subtilis* (B), *Lactobacillus casei* (LB), *Saccharomyces cerevisiae* (SC), and their combination (Mix) demonstrated beneficial effects on broiler performance, antioxidant status, immune-related parameters, and intestinal microbial balance. These positive responses support the potential role of probiotics as functional feed additives capable of improving bird health and productivity through multiple biological pathways that may collectively contribute to improved bird health and productivity. The improvements in growth performance observed in the present study, particularly the significant increases in body weight gain and improvements in feed conversion ratio, may primarily be attributed to enhanced intestinal function and nutrient utilization. Although feed intake was not affected by probiotic supplementation, birds receiving probiotics exhibited greater body weight gain and improved feed efficiency, suggesting improved nutrient utilization efficiency. The improvement in productive performance following probiotic supplementation may be attributed to the ability of probiotics to regulate intestinal microbial communities, enhance nutrient utilization, and maintain intestinal homeostasis^21^. Probiotics can improve intestinal conditions by promoting beneficial microorganisms and limiting the colonization of undesirable bacteria through competitive exclusion^22^. This mechanism involves competition for adhesion sites and available nutrients, production of antimicrobial compounds, and modification of the intestinal environment, which collectively reduce the opportunity for pathogenic microorganisms to establish and multiply^23^. Therefore, improved microbial balance may enhance digestive efficiency and nutrient absorption, contributing to better growth performance and feed conversion efficiency^24,25^. Previous studies have demonstrated that probiotic supplementation improved growth performance, health status, feed efficiency, and intestinal microbiota composition in broiler chickens^24–27^.

The beneficial effects of probiotics on broiler performance may also be related to their ability to produce beneficial metabolites, particularly short-chain fatty acids (SCFAs)^28^. These microbial metabolites serve as energy sources for intestinal epithelial cells, contribute to maintaining intestinal pH, and support intestinal barrier function^29^. A healthy intestinal barrier reduces the translocation of harmful microbial products and improves the efficiency of nutrient utilization, which may partially explain the positive effects of probiotic supplementation observed in the current study. In addition, probiotics may stimulate endogenous digestive processes by enhancing enzyme activity and improving the availability of nutrients for absorption^30^.

*Bacillus subtilis* is considered one of the promising probiotic candidates in poultry nutrition due to its ability to tolerate gastrointestinal conditions and maintain viability during feed processing^31^. The beneficial effects of *Bacillus* supplementation may be associated with its production of extracellular enzymes, antimicrobial peptides, and other bioactive metabolites that contribute to improved digestion and microbial stability within the intestine^32^. By reducing the pressure of harmful microorganisms and supporting beneficial microbial populations, *Bacillus subtilis* can improve intestinal functionality and overall bird performance. Previous studies have reported that *Bacillus subtilis* supplementation enhanced productive performance, immune response, antioxidant status, and gut health in broilers^3,33–36^.

*Lactobacillus casei* may exert its beneficial effects through several complementary mechanisms, including production of organic acids, competitive exclusion of pathogenic bacteria, and enhancement of intestinal microbial balance^37^. The production of lactic acid can decrease intestinal pH, creating an unfavorable environment for many harmful bacteria, while simultaneously supporting the growth of beneficial microorganisms^38^. Moreover, *Lactobacillus* strains can improve intestinal barrier integrity and contribute to more efficient nutrient utilization, which may explain their positive effects on broiler productivity^38^. These mechanisms are consistent with previous reports indicating beneficial effects of probiotic *Lactobacillus* supplementation on poultry health and performance^37,39^.

*Saccharomyces cerevisiae* has also been recognized as an effective probiotic feed additive due to its ability to improve intestinal health, nutrient utilization, and immune function in poultry^40^. The beneficial effects of yeast supplementation may be associated with the presence of bioactive components, including β-glucans and mannan-containing compounds, which can interact with the intestinal environment and support host defense mechanisms^40^. Yeast components may contribute to the reduction of undesirable bacterial attachment to intestinal epithelial surfaces and promote a more favorable microbial ecosystem^41^. Furthermore, *Saccharomyces cerevisiae* may enhance digestive activity and nutrient availability, leading to improved growth performance and physiological status of broiler chickens^42^. Previous studies have demonstrated that yeast supplementation positively influenced productive performance, intestinal microbiota, intestinal integrity, immune status, and nutrient utilization in broilers^42–45^.

The improvement observed in birds receiving the probiotic mixture (Mix) may be explained by the synergistic and complementary actions among *Bacillus subtilis*, *Lactobacillus casei*, and *Saccharomyces cerevisiae*. Each probiotic strain may contribute through different but interconnected mechanisms. *Bacillus subtilis* can provide antimicrobial activity and support digestion through enzyme production, *Lactobacillus casei* can regulate intestinal microbial balance through organic acid production and competitive exclusion, while *Saccharomyces cerevisiae* can enhance intestinal integrity and immune modulation. Therefore, combining these microorganisms may provide a broader spectrum of beneficial effects compared with individual supplementation by targeting different aspects of intestinal function and host physiology. The synergistic interaction among probiotic strains may explain the superior response observed with the Mix treatment. In agreement with this concept, previous findings showed that supplementation with *Bacillus subtilis*. and *Saccharomyces cerevisiae* improved broiler performance, beneficial bacterial populations, antioxidant status, intestinal parameters, and immune responses^43^.

The absence of detectable Salmonella and the lower total bacterial count observed in the Mix group may be attributed to the complementary activities of the tested probiotics, including competitive exclusion of pathogens, competition for intestinal adhesion sites, and the production of antimicrobial metabolites and organic acids, which collectively may contribute to a more favorable intestinal microbial environment.

The improvement in immune-related blood parameters following probiotic supplementation may be associated with the immunomodulatory properties of probiotics. Probiotics can interact with intestinal immune cells and stimulate innate and adaptive immune responses through microbial-derived signals^46^. In particular, beneficial microorganisms and their metabolites can enhance immune system development, improve protein metabolism, and support the production of immune-related proteins^46^. The improvement in total protein, albumin, globulin, and albumin/globulin ratio observed following probiotic supplementation indicates enhanced physiological and immune status of broilers^42,47^. Previous studies have suggested that probiotics can enhance antioxidant and immunological status of broiler chicks^33,42^.

The absence of adverse effects on liver, kidney, and bone function indicators indicates the safety of the tested probiotic treatments and confirms their suitability as feed additives for broiler production. Maintaining normal biochemical parameters suggests that probiotic supplementation did not induce metabolic stress or organ dysfunction. Instead, the improved physiological status observed in supplemented birds may be associated with better intestinal function, improved nutrient utilization, and reduced microbial challenges.

The antioxidant improvement observed following supplementation with B, LB, SC, and Mix may be related to the ability of probiotics to regulate oxidative balance and enhance the endogenous antioxidant defense system. Rapid growth in broilers is commonly associated with increased metabolic activity and reactive oxygen species (ROS) generation; therefore, maintaining an efficient antioxidant system is essential for protecting tissues against oxidative damage. Probiotics may reduce oxidative stress by decreasing harmful microbial metabolites, improving intestinal barrier function, and stimulating antioxidant enzymes such as catalase. The reduction in lipid peroxidation and improvement of antioxidant capacity indicate enhanced protection of cellular components against oxidative injury. These findings are consistent with previous reports indicating that probiotics improve antioxidant and physiological status in broiler chickens^33,36,39,42^.

The improvement in intestinal microbial balance observed following probiotic supplementation provides further evidence for the beneficial role of probiotics in maintaining gut health. The increase in *Lactobacillus* populations and reduction in total bacterial counts indicate that probiotic supplementation promoted a more favorable intestinal microbial environment. This beneficial modulation of gut microbiota may occur through several mechanisms, including competitive exclusion of harmful microorganisms, production of organic acids and antimicrobial compounds, and enhancement of intestinal ecological stability^48^. By increasing beneficial bacterial populations, probiotics may contribute to improved intestinal function and more efficient nutrient utilization.

**Fig. 6:**
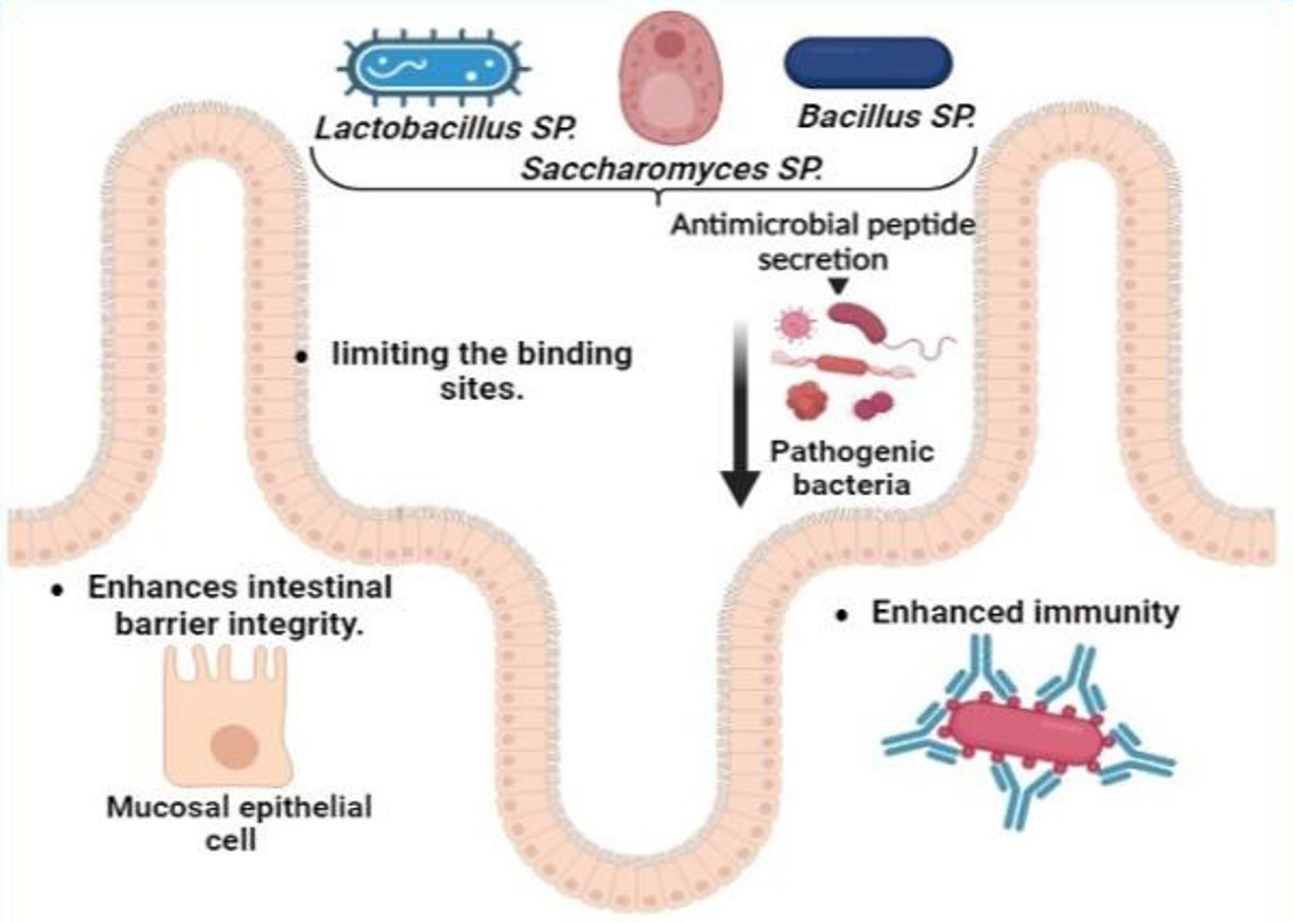
Illustrates the mechanism by which probiotics replace antibiotics in promoting growth.

The ability of probiotics to limit harmful microorganisms represents one of the most important mechanisms explaining their role as alternatives to antibiotic growth promoters. Probiotics can prevent pathogen establishment by competing for intestinal adhesion sites, producing antimicrobial substances, and modifying the intestinal environment. In addition, probiotics may enhance intestinal barrier integrity by supporting epithelial cell function and regulating immune responses. These mechanisms collectively contribute to maintaining intestinal health and improving growth performance without the need for antibiotic supplementation. As illustrated in Fig. 6, the mode of action of probiotics involves multiple pathways, including microbial competition, antimicrobial activity, intestinal barrier enhancement, and immune modulation^49–51^.

Although the present study demonstrated beneficial effects of probiotics on broiler performance, antioxidant status, biochemical parameters, and intestinal microbial balance, some limitations should be considered. The absence of an antibiotic positive control group represents a limitation because direct comparison between probiotic supplementation and conventional antibiotic growth promoters was not performed. Therefore, future studies including an antibiotic-treated group would provide further evidence regarding the relative efficacy of probiotics as antibiotic alternatives. In addition, intestinal histomorphological evaluation, including villus height, crypt depth, and villus-to-crypt ratio, was not conducted in the current experiment. Such measurements would provide additional information regarding the effects of probiotics on intestinal structural development and nutrient absorption capacity.

In conclusion, dietary supplementation with *Bacillus subtilis*, *Lactobacillus casei*, *Saccharomyces cerevisiae*, and their combination improved broiler productive performance, antioxidant status, immune-related parameters, and intestinal microbial balance without causing adverse effects on liver, kidney, or thyroid functions. The beneficial effects of probiotics may be attributed to their multiple biological activities, including competitive exclusion of harmful microorganisms, production of beneficial metabolites, enhancement of intestinal barrier function, stimulation of antioxidant defense mechanisms, and modulation of host immunity. The complementary effects of combined probiotic supplementation suggest that multi-strain probiotic formulations may represent a promising natural strategy for improving poultry production and reducing dependence on antibiotic growth promoters.

## Data Availability

The datasets used and/or analyzed during the current study available from the corresponding author on reasonable request.
